# Network analysis investigating the differentiation of achievement goal orientations in junior high school

**DOI:** 10.12688/f1000research.167284.1

**Published:** 2025-11-10

**Authors:** Georgia Stavropoulou, Maria Gkevrou, Dimitrios Stamovlasis

**Affiliations:** 1 Philosophy and Education, Aristoteleio Panepistemio Thessalonikes Philosophike Schole, Thessaloniki, Makedonia Thraki, Greece

**Keywords:** achievement goal orientations, perceived teachers goals, junior high school, network analysis, multiple goals

## Abstract

Achievement goal orientation theory has become popular, as it interprets students’ academic attitudes and behavior. The present research aims to investigate the variation in teachers’ perceived goals in relation to achievement goals in different grades, as well as the emergence of the multiple goal theory. Participants were junior high school students who responded to a self-report questionnaire. The instrument used was the Patterns of Adaptive Learning Scales (PALS). The results revealed that mastery and performance goals behave as a network of interacting variables that essentially represent their coexistence, as well as their individual variations and specificities in relation to other variables. However, a significant distinction between mastery goals and perceived mastery goals with performance goals and perceived performance goals became apparent for each individual grade. The present research contributes to the theoretical development of the field, while in practice it highlights the combination of goals in school reality.

## Introduction

### Achievement goal orientations

Goal orientation theory provides a framework and psychological constructs to explain learning behaviors and outcomes. Initially, goals were divided into mastery and achievement goals ( Dweck & Leggett, 1988; Ames, 1992) until Elliot and Harackiewicz (1996) presented a trichotomous model that included mastery, performance-approach, and performance-avoidance goals. Subsequently, performance-avoidance goals were proposed as an additional dimension. However, the resulting 2 × 2 model is not well supported in the literature ( Barron & Harackiewicz, 2001, 2003). For these reasons, the present study used the developed three-dimensional model. The three goal orientations associated with different perceptions of success explain students’ academic behavior. The mastery goal is associated with learning patterns that emphasize deeper understanding and learning; therefore, it is related to adaptive behavioral patterns such as high self-efficacy, strong interest in school, use of strategies, and emotional and behavioral engagement (e.g., Elliot & Hulleman, 2017; Gonida et al., 2009; Senko, 2019; Scherrer et al., 2020; Stavropoulou et al., 2023, 2024). In contrast, performance-avoidance goals are associated with maladaptive (negative) norms, whereas performance-approach goals are associated with both adaptive and maladaptive (e.g., Linnenbrink, 2005; Senko & Dawson, 2017).

In addition to individual achievement goals, a different perspective of the theory was proposed: that of multiple goals. In the school environment, students may adopt two or more goals simultaneously. In this case, each goal has its own effect and students are led to different academic outcomes each time. Four types of multiple goals have been proposed: the *additive* model, *specialized* model, *selective* model, and *interaction* of two or more objectives ( Barron & Harackiewicz, 2001, 2003). The most constructive combination is considered to be the one that combines high mastery goals and performance-approach goals, as students can gain benefits from both ( Barron & Harackiewicz, 2001; James & Yates, 2007).

Goal orientation theory has been widely used in educational contexts, from primary schools to educational institutions in higher education (e.g., Stavropoulou et al., 2024; Stavropoulou & Stamovlasis, 2024, 2025a, b). The trichotomous model of goal orientation theory plays an important role among adolescents. Adolescents often face difficulties in their transition to secondary education as they are more vulnerable to change. Adolescents are vulnerable during the transition to secondary education because psychological and hormonal changes, increased academic demands, social adjustments, loss of familiar support systems, increased autonomy, and pressure from parents and society can all contribute to stress and uncertainty. Specifically, when students reach secondary school, they experience more distant relationships with teachers, which leads to negative outcomes, such as dropout or lower achievement (e.g., Henry et al., 2011), academic disengagement ( Galvan et al., 2011), and decreased motivation. Adolescents often perceive the academic environment to be more performance-oriented ( Midgley & Urdan, 1995). It is worth mentioning that from ages to 8-15 years, decreased intrinsic motivation has been observed ( Lepper et al., 2005), whereas after 15 years of age, an increase is observed because students are oriented towards tasks that are of interest to their future careers ( Gillet et al., 2012; Gottfried et al., 2001). Moreover, it has been shown that adopting the performance-approach goal does not always lead to better performance in high school students; however, lowering mastery goals leads to negative outcomes such as lower self-efficacy, while increasing them leads to more adaptive learning patterns (e.g., Paulick et al., 2013).

### Perceived teachers’ goals

Among other factors, classroom climate and school context play an important role for students; in particular, how they perceive the academic environment and the goal orientations it promotes. Their interpretation of stimuli is linked to their emotions and behavior in an academic context ( Anderman & Maehr, 1994; Maehr & Midgley, 1991). Research has highlighted the predictive role of perceived goals in academic behavior, with students’ goal orientations acting as mediators ( Kaplan & Maehr, 1999). This strong effect of perceived goals is explained by their association with motivation (e.g., Ryan & Patrick, 2001; Gonida et al., 2009).

Teachers and their effective practices play a key role in the classroom climate that promotes certain goal orientations that are potentially adopted by students, influencing their cognitive outcomes and motivation ( Bardach et al., 2020; Maulana et al., 2016). This valuable ability of teachers to motivate their students seems to be prevalent in relevant research and correlates with other vital factors, such as resilience, which acts as a mediator in school performance ( Escalante Mateos et al., 2021).

Looking at specific goal orientations, research has revealed a strong relationship between mastery goals and performance-approach goals (e.g., Gonida et al., 2009; Bardach et al., 2020; Urdan & Schoenfelder, 2006). In contrast, for performance-approach and performance-avoidance goals, the findings are contradictory. Studies have shown that there is a negative relationship between performance-avoidance structural goals and performance-approach goals (e.g., Kim et al., 2010), while others have reported a positive correlation between performance-approach goals and mastery goals (e.g, Lüftenegger et al., 2017). However, no such relationship has been found between mastery goal orientations and performance-approach goals (e.g., Bardach et al., 2020; Schwinger & Stiensmeier-Pelster, 2011). However, the relationship between performance-approach and performance-avoidance has been established and found to be more pronounced in secondary education ( Bardach et al., 2020).

### Self-efficacy

Self-efficacy plays a vital role in the learning process and acts as a catalyst and a predictive factor. Based on the latest empirically supported theoretical framework, students focused on mastery tend to possess elevated levels of self-efficacy (e.g., Lüftenegger et al., 2017; Stavropoulou et al., 2023; Stavropoulou & Stamovlasis, 2024). Their actions align with the findings in the existing literature, showing traits such as increased optimism, a positive approach to learning, and a proactive stance in learning from errors by employing strategies ( Friedel et al., 2007; Senko & Dawson, 2017; Tuominen-Soini et al., 2012). Moreover, research has demonstrated that a reduction in mastery goals is linked to adverse effects, such as decreased self-efficacy, whereas an upsurge in mastery goals is associated with more advantageous patterns ( Paulick et al., 2013). As previously noted, performance goals can have varying positive, negative, or negligible ( Hulleman et al., 2010). Furthermore, they may be associated with elevated self-efficacy, the application of profound strategies, and strong performance ( Pintrich, 2000), or they could be linked to superficial strategies and the choice of simpler tasks ( Sideridis & Stamovlasis, 2016; Senko & Dawson, 2017) and no relationship has been proven with performance goals ( Diseth et al., 2012; Stavropoulou et al., 2023).

Studies have also revealed that perceived self-efficacy is among the strongest predictors of motivation and performance in writing ( Bruning & Kauffman, 2015), because it connects to important factors such as the perceived importance of writing, comprehension, and use of strategies ( Graham & Harris, 2000). For example, when students engage in writing and regard it as an essential skill, they typically adopt a mastery-goal orientation (e.g., Robins & Pals, 2002) and which could potentially boost their self-efficacy (e.g., Limpo & Alves, 2017; Pajares, 2003). The connection between self-efficacy and writing performance goals has not been definitively identified ( Kaplan et al., 2009). Nevertheless, there is some indication of a negative association between self-efficacy and performance-avoidance goals, along with a positive association with performance-approach goals, as reported in studies (e.g., Pajares et al., 2000).

### Purpose and research questions

This study aimed to investigate the changes and variations in both individual achievement goals and perceived goals in junior high school. The research questions were as follows.

1) How is the network of multiple goals organized and structured, and which nodes seem to be the most influential in the network, based on measures of centrality in the three junior high school grades?
2) Is there any differentiation of individual achievement goals and perceived achievement goals from 7th to 9th grade?
3) To which achievement goals are self-efficacy beliefs most closely associated?
4) Does the present networks behave as small world networks?

### Rationale-methodological considerations

This study adopts a non-linear approach to explore the relationships among motivation variables, focusing on mastery and performance goals, along with perceived teachers’ goals. Unlike traditional methods ( Linnenbrink et al., 2018; Stavropoulou et al., 2023), it applies network ontology to represent these psychological constructs as systems of interconnected elementary concepts, which is consistent with complexity theory. This approach draws on latent-variable representation theories and uses network analysis to reveal emergent qualitative entities. This methodology aligns with contemporary psychometric frameworks, highlighting the innovative use of network analysis in studying motivational goals ( Borgatti & Halgin, 2011; Siew, 2020; Siew et al., 2019).

Network analysis, which is a key component of complexity theory, employs mathematical methods to study the internal structures of complex systems. It simplifies systems by focusing on the active components and their interactions and removing unnecessary details. Using mathematical graph theory, it defines and analyzes system boundaries, components, and relationships, providing a systematic approach for understanding complex domains ( Koponen & Nousiainen, 2014).

Network ontology is rooted in the meta-theoretical framework of complexity science, which views systems as being composed of many interconnected and co-evolving parts ( Stamovlasis, 2016; Stamovlasis & Koopmans, 2014). Network science provides mathematical tools for studying these systems, representing their internal structures as nodes and links. The complexity theory emphasizes that the properties and behavior of such systems emerge from interactions among elements and as a unified whole. It blurs the distinction between quality and quantity, treating them as interdependent attributes within a unified framework that varies by the complexity level observed, whether micro or macro ( Koopmans & Stamovlasis, 2016; Stamovlasis, 2016a).

Network analysis as part of complexity is an important and emerging field of research that studies complex systems, from biological to social and psychological, focusing on the patterns of relationships between the constituent parts and highlighting the flow of interaction in the system. The structure of complex systems is represented in the form of a web of nodes and connections between nodes. Mathematically, the above approach corresponds to graph analysis, with a significant contribution in shifting the focus from the part to the whole, and provides the methodological tools to abandon reductionism in favor of a review of the whole.

It is appropriate to clarify that network ontology belongs to the meta theoretical framework of complexity science. Complexity theory assumes the ontological characteristics described by networks, that is, many interconnected and interacting parts that co-evolve over time ( Stamovlasis, 2016a; Stamovlasis & Koopmans, 2014). Network science provides a mathematical formulation for studying these complex systems and investigating the internal structure consisting of nodes and links. A key aspect of a complex system is that its properties and behavior are described in terms of the underlying interacting elements as well as in terms of the system as a unit ( Koopmans & Stamovlasis, 2016; Stamovlasis, 2016a, 2016b).

The objective of applying a Network Analysis is not to observe or measure the manifestations of a single underlying attribute. Instead, it is the network of relationships between elements that is considered to constitute the individual differences under investigation. According to this perspective, psychological characteristics exist as a system of interrelated elements. In classical psychometrics, psychological constructs are treated as independent entities that can be measured separately; however, their ontological status remains unclear. By contrast, Network Analysis provides a specific ontological perspective that aligns with both its methodological approach and data analysis. Psychological traits are conceptualized as complex networks of interrelated components, offering an alternative to the classical latent variable approach without necessarily excluding it ( Guyon et al., 2017; Neal et al., 2022). The key difference is that in the classical approach, the latent variable (psychological construct) is viewed as the common cause of empirical indicators, whereas in network theory, causality is not attributed to specific individual variables or hypothetical entities, but rather emerges from the overall configuration of the network, which consists of observed variables (nodes).

Cognitive, mental, and psychological processes are regarded as complex systems that give rise to corresponding behaviors ( Siew et al., 2019). Beyond its application in various fields, network theory provides both conceptual and methodological tools for a deeper understanding of cognitive structures and processes ( Gkevrou & Stamovlasis, 2022; Stella, 2020), although its use in these domains remains relatively limited.

A fundamental requirement for studying psychological or cognitive systems as networks is to meaningfully represent them in terms of nodes and edges. Specifically, nodes and edges in any psychological network should correspond to theoretically justified structures, where nodes provide an appropriate and relevant representation and edges define meaningful relationships between them. The choice of representation often aligns with the measurement instrument because different representations can highlight distinct aspects of the underlying cognitive system. For instance, if a questionnaire is used, the nodes may represent the psychological variables.

Network analysis generally involves two stages: First, researchers estimate and apply appropriate statistical modelssuch as correlation, partial correlation, or linear regressionto the data. The resulting parameters can then be represented as a weighted network of the observed variables. Second, the weighted network was analyzed using graph-theoretical measures to extract key insights, such as identifying central nodes, detecting potential communities, and assessing network structure. This is precisely the function performed by the JASP software.

In psychological networks, the strength of connections between nodes is an estimated parameter derived from empirical data. As the sample size increases, these parameter estimates become more accurate and theoretically approach their true values ( Borsboom et al., 2021; Epskamp et al., 2018).

Once the network is constructed and its structure defined, researchers are encouraged to apply network analysis metrics to explore how the organization of nodes—whether conceptual elements or psychological variables—reflects cognitive structures or belief systems. This approach allows for an examination of how conceptual, affective, cognitive, or attitudinal components are perceived and interconnected ( Stella et al., 2019).

The complexity paradigm has influenced numerous studies in the social sciences, examining shared networks of entities connected through relational links, such as friendship, cooperation, or interaction ( Bruun et al., 2019; Marion & Schreiber, 2016; Tsiotas & Polyzos, 2018). Beyond physical connections, it also encompasses intangible webs such as semantic, linguistic, or psychological networks. Recently, network analysis has gained prominence in identifying social and cognitive patterns across various contexts, including education ( Katerelos & Koulouris, 2004; Nousiainen & Koponen, 2020; Stella, 2020; Sun et al., 2020).

## Μethod

### Sample

In the present study, students attending 7th (N = 543), 8th (N = 502), and 9th grades (N = 297) in junior high school participated. The survey was approved by the Ethics Committee of the Aristotle University of Thessaloniki and in order to collect the sample, the necessary consent forms for parents and guardians were provided. These forms informed both the purpose and procedure of the research. Only students whose parents or guardians consented to participate of their children in this study were included in the survey. Parental consent was obtained through consent forms and was shared digitally in a written way. This study adhered to the guidelines of the Declaration of Helsinki and was approved by the Ministry of Education. This research was approved by the Ethics Committee of the Institute of Educational Policy of Greece (1817/06-03-2018/ΙΕΠ). According to the Regulation of Principles and Operations of the Ethics and Research Integrity Committee of Aristotle University of Thessaloniki (published in July 2020: https://websites.auth.gr/ehde/wp-content/uploads/sites/65/2024/05/Regulation-EHDE-en.pdf), which was drafted in accordance with the provisions of Law 4485/2017, Article 68, and Law 4521/2018, articles 21-27, the mandatory submission for evaluation by the committee applies in the case of funded research projects that do not apply to the present work. However, we confirmed that all the procedures performed in this study followed the guidelines of the Declaration of Helsinki. Any measures for personal data protection were also taken according to the DPO instructions.

### Materials

The instrument used for individual achievement goals mastery goals, (map), performance-approach goals, (perfap), performance-avoidance goals (perfav), and teachers’ perceived goals; especially teachers’ mastery goals, (gsmap), teachers’ performance-approach goals, (gsperfap), and teachers’ performance-avoidance goals, (gsperfav). We also measured the students’ self-efficacy (self ). The instrument used was the revised version of the Adaptive Learning Scale (Adaptive Learning). This study employed the Patterns of Adaptive Learning Surveys (PALS) scale, a trichotomous model developed by Midgley et al. (1998). This scale was chosen over the alternative Achievement Goal Questionnaire (AGQ-R; Elliot & Murayama, 2008) to ensure comparability and reproducibility of the results, as it is the most widely used in Greek research.

### Data analysis

Data analysis was performed using JASP, focusing on the microscopic level of network analysis, which examines individual nodes to comprehensively address research questions. JASP is free and user-friendly statistical software that offers both frequentist and Bayesian analyses, making data analysis accessible to researchers at all levels. Centrality measures include strength, the sum of absolute edge weights directly connected to a node ( Bringmann et al., 2019; Burger et al., 2022; Siew et al., 2019; Siew, 2020); closeness centrality, indicating a node’s connectivity within the network; and betweenness centrality, reflecting a node’s role as a bridge in the shortest paths ( Letina et al., 2019; Siew, 2020). The local clustering coefficient, which assesses the interconnectedness of a node’s neighbors and proximity reciprocity, was also applied ( Siew et al., 2019; Siew, 2020). These measures collectively capture various aspects of node positioning and their influence within the network.

## Results

**Figure 1.**
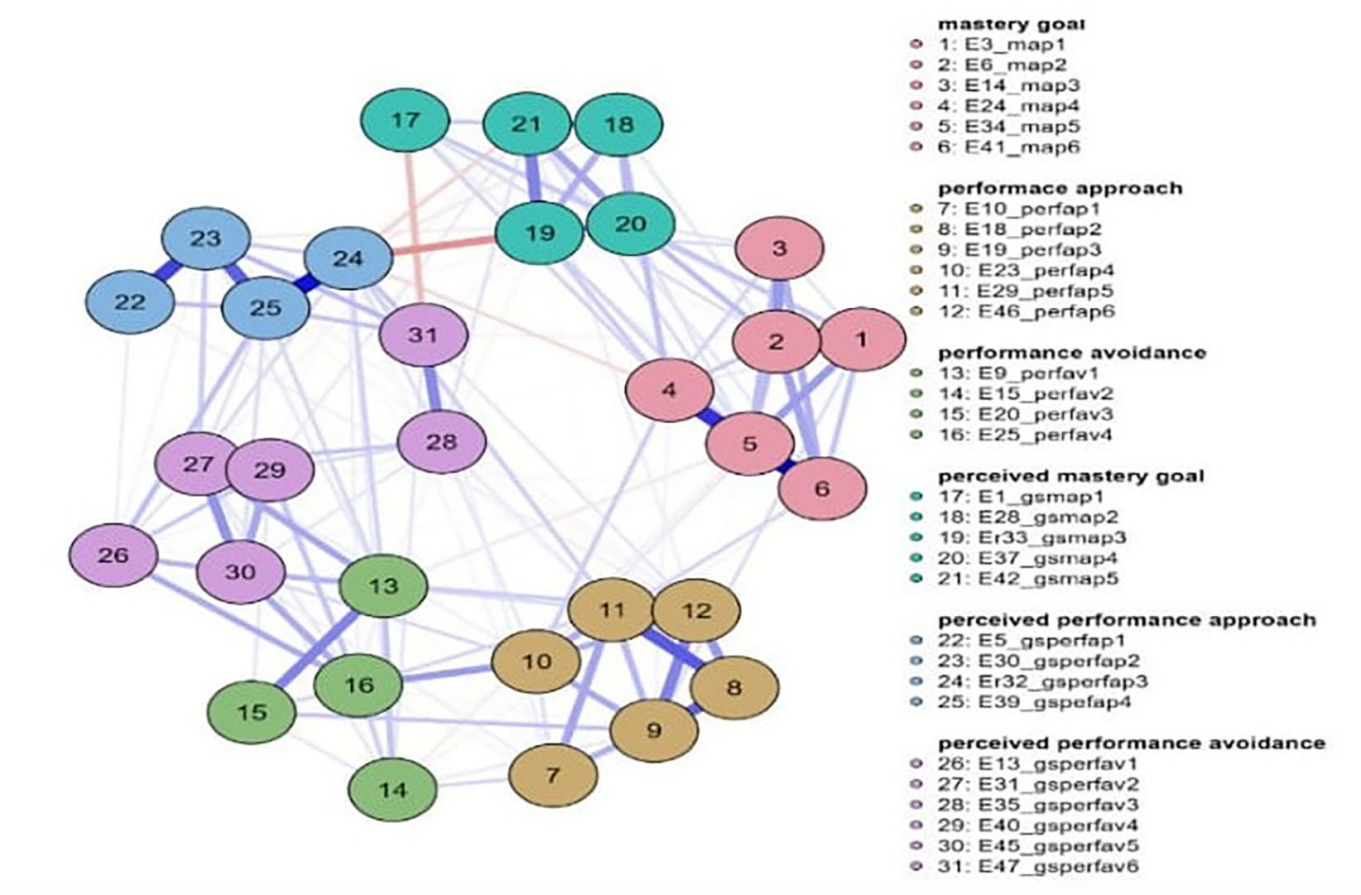
Network for 7th grade.

**Figure 2.**
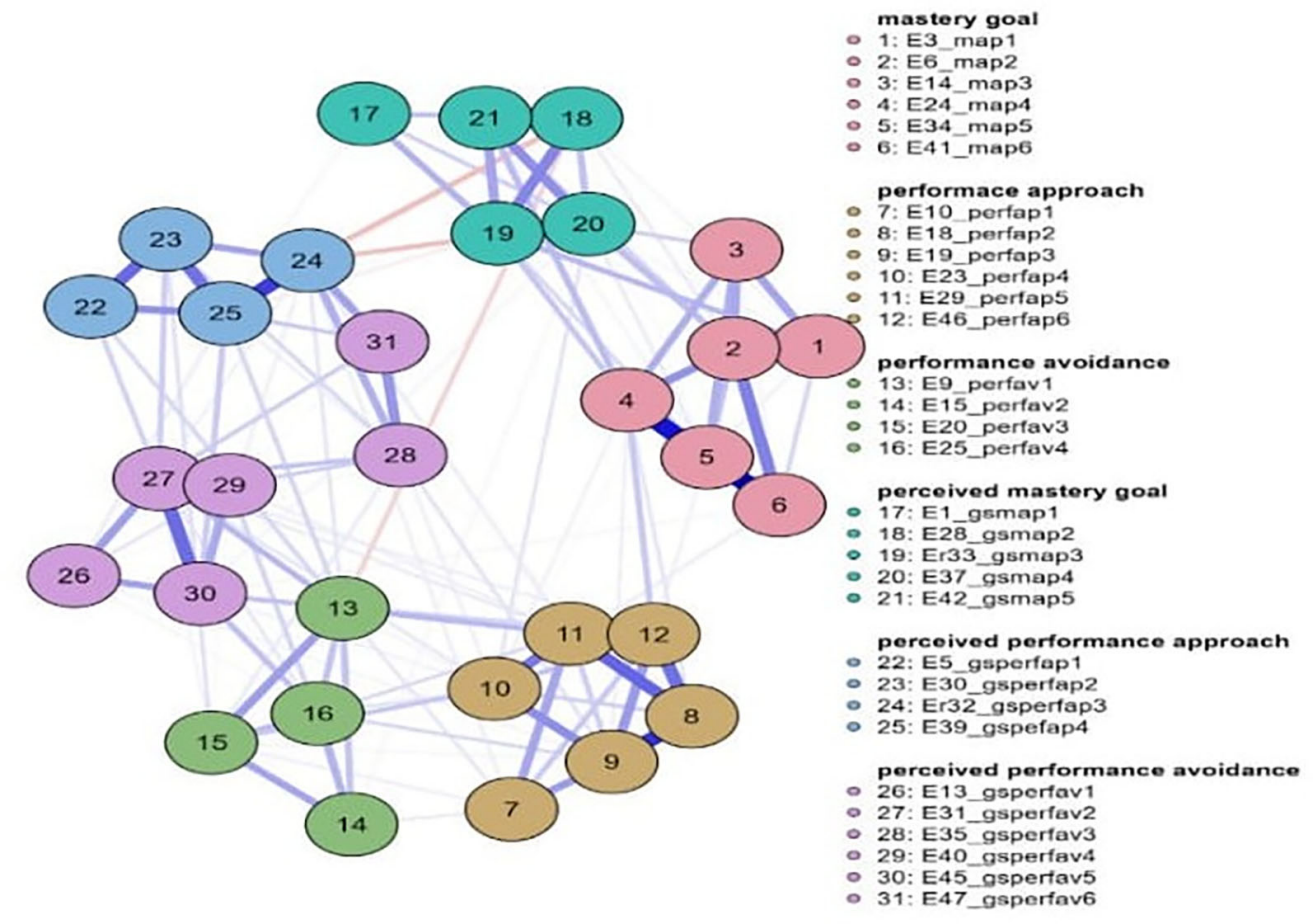
Network for 8th grade.

**Figure 3.**
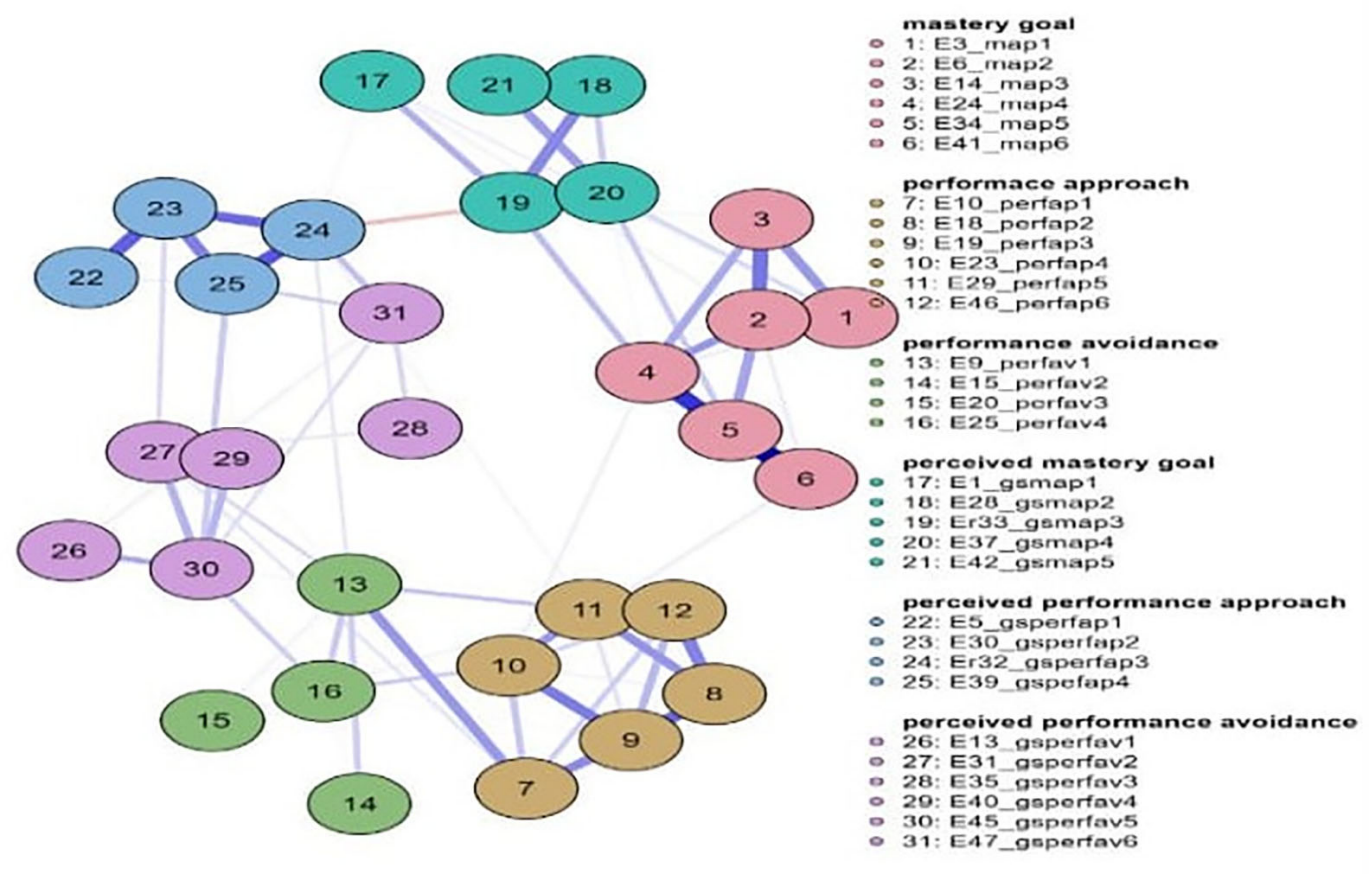
Network for 9th grade.

Three networks emerged from the data analysis, each corresponding to one grade of junior high school ( Figures 1, 2, and 3). The overall diagram of centrality measures is presented ( Figure 1). In more detail, and in terms of the structure of all three networks, with a gradual peak from 7th grade to 9th grade, is the distinction of the networks into two main sub-networks in addition to the individual groupings. More specifically, it appears that mastery goals and perceived mastery goals form one part, while the remaining performance goals and perceived performance goals form the other. This is based on the number and type of connections created between sets of nodes. Few weak and/or negative connections were observed between the nodes corresponding to perceived mastery goals and perceived performance-approach goals. The connections between the mastery goals and the remaining nodes were similar. However, this does not occur for perceived mastery goals.

A similar change was observed in the centrality measures examined in this analysis. Specifically, in the network of the 7th graders in the measure of strength, the nodes that emerged as the most significant variables were map 5 = 2.057, gsperfap3=1.700, perfap5 = 1.414, and gspefav2 = 1.344. Betweenness highlighted the nodes: gsperfap3 = 3.504, gsmap3 = 1.713, gspefap4 = 1.387, and perfap4 = 1.346. Concerning closeness, the strongest nodes were gsperfap3 = 2.749 and gsperfap4 = 2.073 gsmap3=1.633. This information is presented in Figure 1.

Concerning 8th grade ( Figure 2), the network nodes with the highest strength are gsperfav2 = 2.020, perfap5 = 1.534, and map 5 = 1.202. The following nodes are highlighted in the measure of betweenness: perfav1 = 2.439, map 4 = 2.141, perfap6 = 1.962, and perfap5 = 1.724. Regarding closeness, the most important nodes were perfav1=2.340, gsmap2 = 1.478, and perfap6 = 1.313 ( Figure 2).

**Figure 4.**
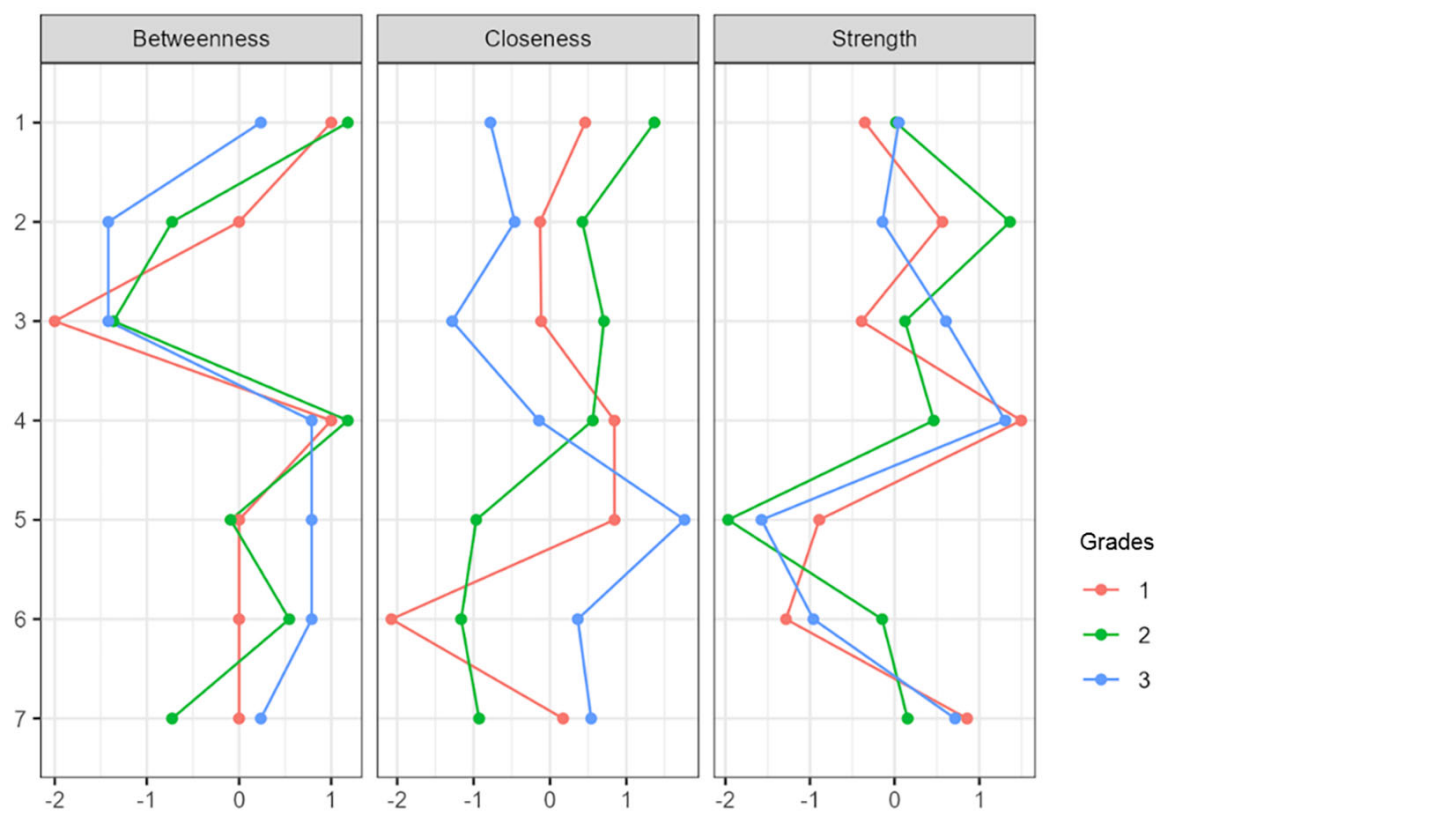
Centrality measures.

Concerning strength in the 9th grade ( Figure 3), the most important nodes were map 5 = 1.562, map 2 = 1.339, and map 4 = 1.242. Nodes gsperfap3 = 3.106, gsmap4 = 2.343, and perfav1 = 1.512 emerged as the strongest in betweenness, while in closeness, the most important nodes were gsperfap3 = 2.048, gsperfap4 = 1.479, gsperfap2 = 1.261, and gsmap4 = 1.233 ( Figure 3). The centrality measures are presented in Figure 4.

**Figure 5.**
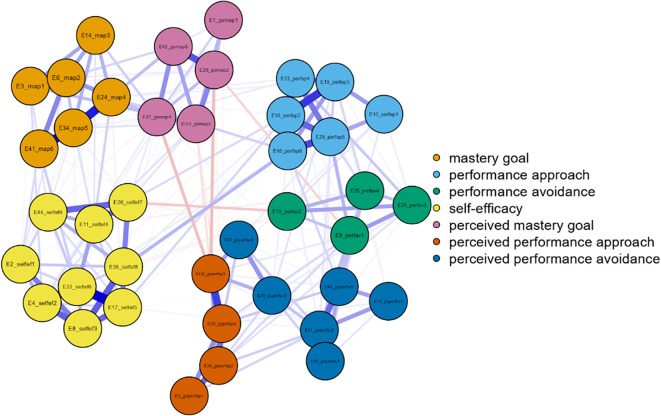
Network of individual and perceived goals with self-efficacy in the 7th grade.

**Figure 6.**
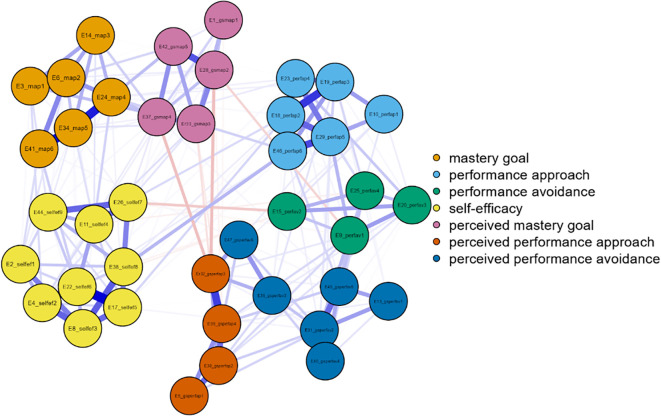
Network of individual and perceived goals with self-efficacy in the 8th grade.

**Figure 7.**
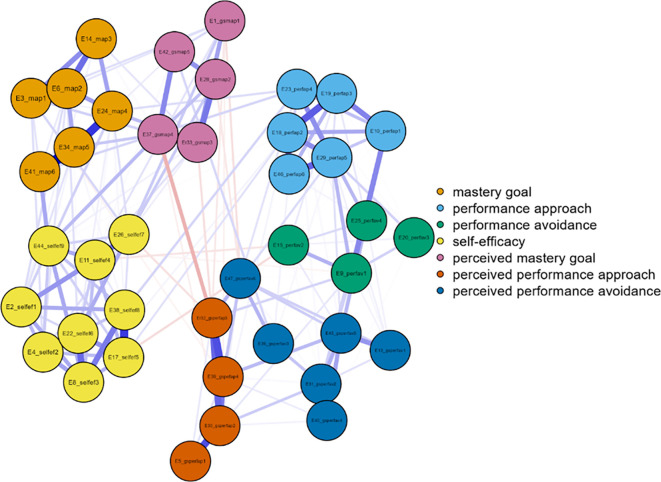
Network of individual and perceived goals with self-efficacy in the 9th grade.

The following networks ( Figures 5, 6 and 7) comprehensively examine the centrality of the node variables under investigation: More specifically, in the network of the 7th grade ( Figure 5) in terms of strength, the nodes that emerged as the most significant were map 5 = 2.077, selfef6 = 1.800, selfef8 = 1.538, gsperfap3 = 1.343, and perfap = 1.225. Betweenness revealed that gsperfap3 = 3.711, gsmap3 = 2.680, gsperfap4, and perfap = 1.172, while gsperfap3 = 2.031, gsmap3 = 1.924, perfap5 = 1.413, and gsperfap4 = 1.250 emerged as the strongest.

In the 8th grade network ( Figure 6), the nodes evaluated as most significant in the strength measure were gsperfav2 = 1.802, selfef6 = 1.673, selfef5 = 1.265, perfap5 = 1.255, and selfef8 = 1.232. The betweenness calculation estimated the most influential nodes to be perfap6 = 3.329, perfav1 = 2.096, map 4 = 1.863, perfap5 = 1.724, and selfef8 = 1.631. Similarly, closeness revealed similar results to the intermediate one, making perfap6 = 2.560, perfap5 = 2.196, and perfav1 = 2.086 the most influential nodes.

**Figure 8.**
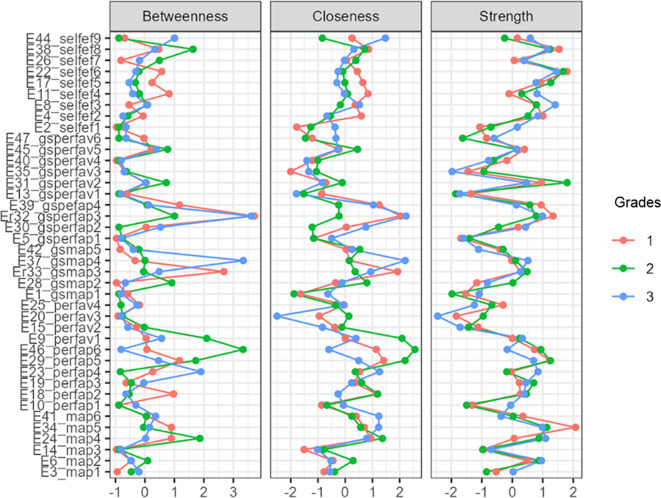
Centrality measures.

Finally, the nodes selfef6 = 1.446, selfef2 = 1.411, selfef8 = 1.160, and map 4 = 1.093 emerged as the most significant nodes in strength in the network of the 9th grade ( Figure 7). In the betweenness measure, nodes gsperfap3 = 3.606, gsmap4 = 3.336, perfap4 = 1.899, and selfef8 = 1.004 emerged as centralities, whereas in the closeness measure, nodes gsperfap3 = 2.235, gsmap4 = 2.201, selfef9 = 1.475, perfap4 = 1.260, map 6 = 1.236, and map 5 = 1.226 emerged as centralities. Figure 8 represents the centrality measures ( Figure 7).

**Figure 9.**
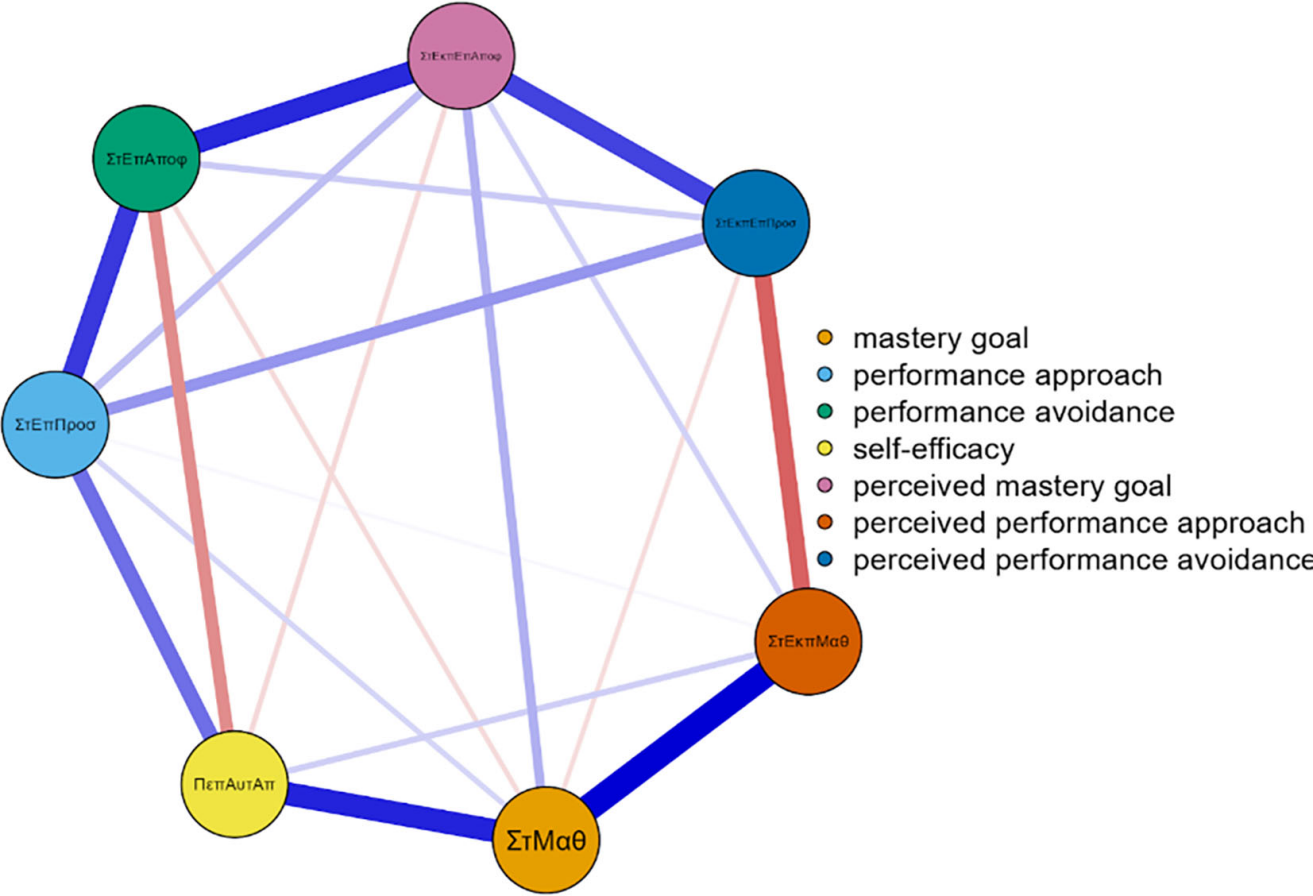
Total network for 7th grade.

To achieve a more global perspective of the above networks, the averages of the variables were used to reform the networks. Consequently, in the 7th grade network, the node of the mastery goals variable is estimated to be the strongest in the measure of strength. Betweenness estimates belonging to mastery goals and self-efficacy beliefs as more critical, while proximity centrality again highlights mastery goals ( Figure 9).

**Figure 10.**
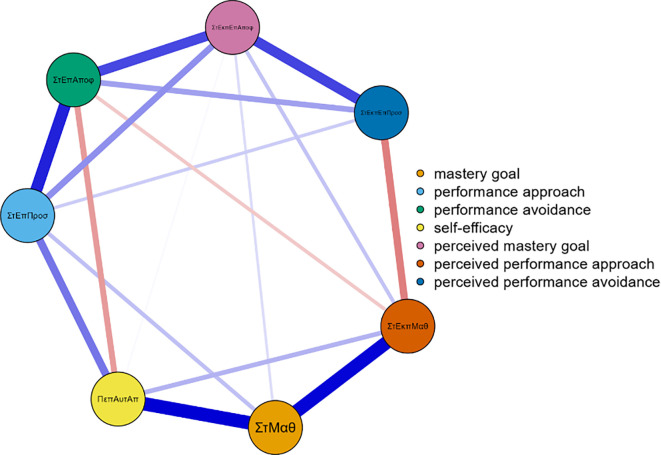
Total network for 8th grade.

The measure of strength in 8th grade’s network estimates the performance-avoidance goals variable as the most central, while mastery goals and self-efficacy beliefs emerge equally in betweenness, which emerges as a stronger node in closeness as well ( Figure 10).

**Figure 11.**
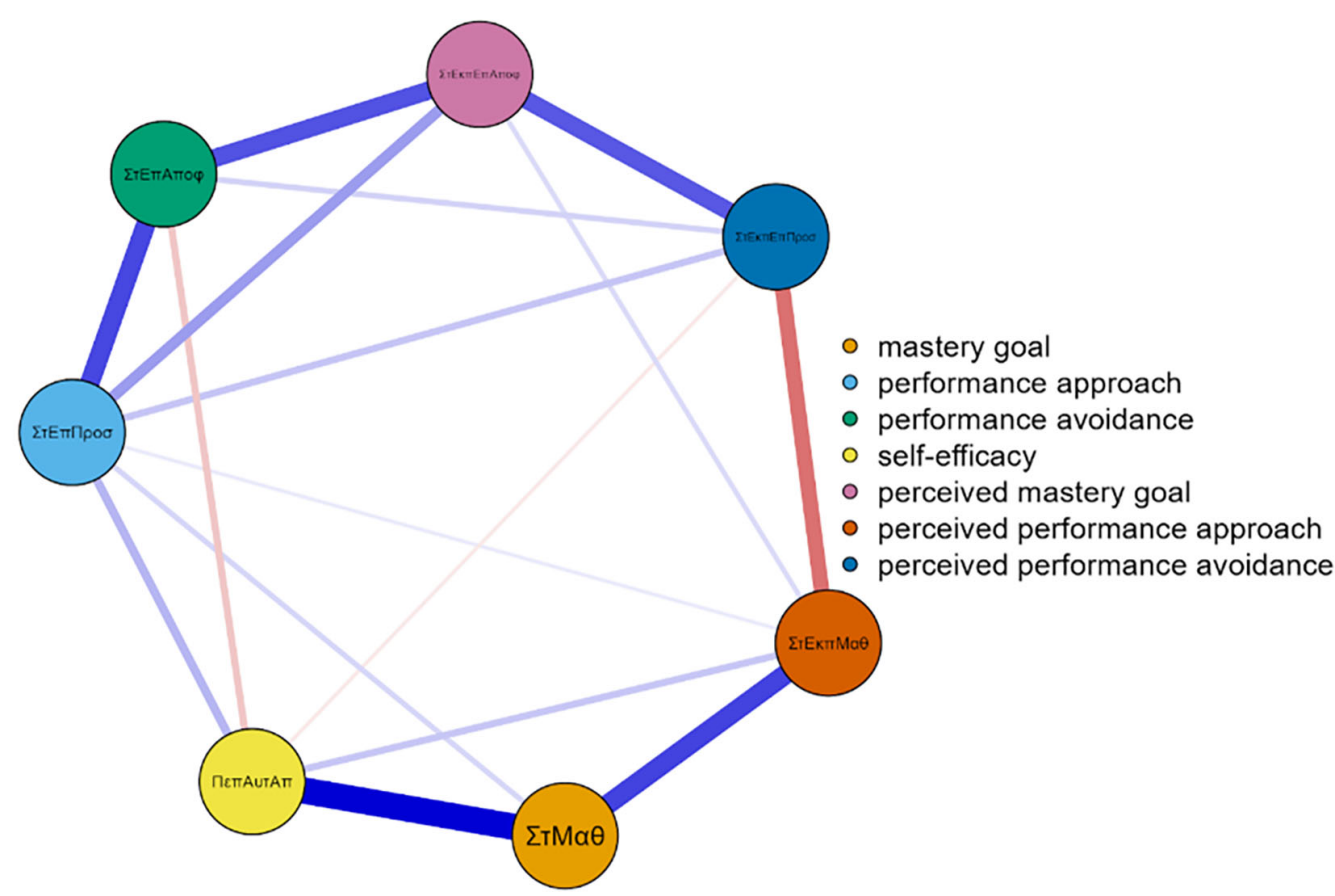
Total network for 9th grade.

**Figure 12.**
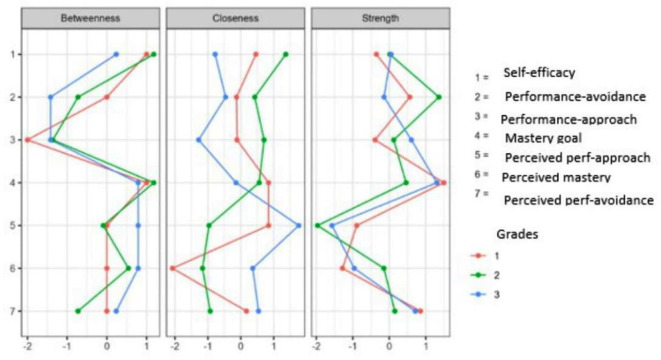
Centrality measures.

Finally, in the 9th grade’s network, the measure of strength highlighted mastery goals as the strongest node. However, the assessment of betweenness indicated that three out of the seven variables were the most important and equal: mastery goals, perceived mastery goals, and perceived performance-approach goals, while only perceived performance-approach goals stood out in closeness ( Figure 11). The centrality measures are represented in Figure 12.

## Discussion

This research led to interesting findings, which are presented in this section. The findings reveal how mastery and performance goals function and are structured within a network of interrelated variables. This network essentially portrays how they coexist, while also highlighting their unique variations and distinct characteristics concerning other variables, including perceived mastery and performance goals, supporting the framework of multiple goals ( Barron & Harackiewicz, 2001, 2003). The first research question concerned how the network of multiple goals is organized and structured, and which nodes seem to be the most influential in the three junior high school grades. Specifically, in the 7th grade perceived performance-approach goals were highlighted as the most significant. In the 8th grade, performance-approach goals were the most significant nodes, whereas in the 9th grade, the most important nodes were perceived performance-approach goals. We observe that in the first and third grades, the most important nodes are ‘perceived performance-approach goals,’ suggesting that students are significantly influenced by the goals that they believe their teachers have adopted. At the same time, in 8th grade, it becomes evident that performance-approach goals play the strongest role, indicating that students are influenced by the goals they perceive their teachers to promote and subsequently adopt ( Friedel et al., 2007). The emphasis on performance-approach goals may be attributed to Greece’s examination system, which strongly focuses on secondary school examinations as the criteria for grade promotion, ultimately aiming for university admission. Afterwards, self-efficacy was added to the network of achievement goal orientations to determine if it influenced the relationship among the nodes. In all three grades, self-efficacy was the most significant variable in the nodes. As it appears, self-efficacy beliefs, when added to the networks, show a completely different picture. Self-efficacy beliefs play a catalytic role because they are associated with all three categories of goals, with different effects on each of them ( Elliot & Hulleman, 2017; Paulick et al., 2013). According to the literature, mastery-oriented students show high self-efficacy, whereas findings related to performance-approach goals with self-efficacy are inconsistent. Therefore, our findings highlight the relationship between mastery-goals and self-efficacy, and performance-approach goals and self-efficacy.

Our next attempt was to evaluate the relationships between achievement goal orientations, perceived teachers’ goals, and self-efficacy. In 7th grade perceived performance-approach was highlighted as the most important node, in 8th grade performance-approach goals were shown as the most significant and in 9th self-efficacy was shown as the most influential node. In this case, it appears to repeat what we observed in the first network concerning individual achievement goals. Consequently, when we examined the overall networks based on the average scores of the variables under investigation, we noticed that in the 7th grade’s network, the predominant node depicted mastery goals. In the 8th grade, it appeared that both mastery goals and self-efficacy beliefs played a decisive role, while in the 9th grade’s network, mastery goals and performance-approach goals were the most important. These points underscore the significance of multiple goals, with mastery goals appearing to be particularly noteworthy ( Barron & Harackiewicz, 2001; James & Yates, 2007).

In general, it is conjectured that most real-world networks follow certain topological and statistical characteristics such as the small-world property. The small-world property, as a macro-structural attribute, describes that the average distance between nodes in a network is relatively shorter than other types of networks, for example, random networks of the same size ( Hwang, et al., 2006). In other words, they are characterized by relatively high levels of transitivity, and nodes are connected to each other through short average path lengths ( Watts & Strogatz, 1998; Hevey, 2018). The existence of this property could be assumed topologically and only in the case of networks of averaged variables ( Figures 7, 8, and 9). It seems, therefore, that the “six degrees of dimension” principle, proposed by Milgram (1967), combined with the formation of closed triangles with respect to the total number of triangles possible for the given number of nodes in the network ( Siew, 2020), is also found here, pointing to a high clustering coefficient, and advocating the hypothesis of the “small world” phenomenon.

In the context of networks of psychometric variables, estimating the small world of these networks can provide an indication of the effectiveness and navigability of students’ internal structures. Macro-level measures can be used to compare the structure of networks before and after educational interventions. Evidence of a successful educational intervention may be reflected in the overall changes in the knowledge network that improve its overall effectiveness and navigability ( Siew, 2020). In general, small-world networks may indicate the presence of hubs. Hubs have central locations in a network and can therefore be ideal for targeted intervention in terms of deliberate change. It has been found that these nodes as hubs are important determinants of survival in network disturbances. These networks are very resistant to accidental attacks, but very vulnerable to targeted “attacks,” thus favoring the effectiveness of a specific educational intervention ( Hwang et al., 2006; Siew, et al., 2019; Siew, 2020).

This discussion highlights that psychometric variables are not static or caused by a single factor, but result from complex interactions involving multiple factors across various levels of description—sociocultural, educational, and psychological. These interactions occur within a framework of circular causality, linking different functions (e.g., psychological structures and perceptions) and operating across various timeframes ( Bolis et al., 2017). This perspective emphasizes that emergent macroscopic structures govern the system as a whole and subordinate the microscopic components that constitute them.

This study argues that by identifying influential nodes and their strongest connections within a network, it is possible to reveal the co-modulation and bidirectional causality between key variables, such as mastery goals and self-efficacy. Indirect communities detected in the network showed connections such as perceived mastery goals with mastery goals, self-efficacy with mastery goals, performance approach, and avoidance with perceived performance goals. These findings are supported by prior research ( Elliot & Hulleman, 2017; Friedel et al., 2007), suggesting that the relationships between these variables are dynamic and interconnected.

Regarding the second research question, which pertains to the differentiation of individual and perceived achievement goals in the three grades, differentiation is observed in 8th grade across all tested combinations of variables. Further details regarding this differentiation are provided in the first section. The overall reshaping of the micro-level network structure may be due to the transition of both the developmental and grade levels. Nevertheless, this finding deserves further investigation.

### Limitations

This study has some limitations. First, the sample size was a major limitation, as there was a larger sample of students in the 7th and 8th grades and a smaller sample in the 9th grade. Another limitation was that the instruments used were self-report questionnaires. Self-reporting instruments have several drawbacks ( Stamovlasis, 2016). Nevertheless, the data collection was conducted at a given point in time (cross-sectional study), with all the disadvantages that this can have. A further limitation is that no emphasis was placed on the school context, according to which the variables under investigation were formed.

### Practical implication

The findings of the present research are quite useful for school reality, as they can explain the behavior of both students and their teachers. These findings can be used by teachers, school psychologists, and/or school counselors to adapt their teaching or interventions and enhance their learning outcomes. Overall, the present research, especially the network analysis method, can be a useful tool in the hands of teachers, helping them to assess the cognitive and other structures of their students and to design their teaching practice to improve their attitudes in the educational context.

## Data Availability

Data is available in the open science framework. Stavropoulou, G., Gkevrou, M., & Stamovlasis, D. (2025a, July 17). Network analysis investigating the differentiation of achievement goal orientations in junior high school. DOI: 10.17605/OSF.IO/84VBX. Data are available under the terms of the Creative Commons Zero “No rights reserved” data waiver (CC0 1.0 Public domain dedication). Study-Specific Approval by the appropriate ethics committee for research involving humans: The research project was approved by the Ethics Committee of the Institute of Educational Policy of Greece (Research Section). Εntrance permission to the schools was provided by the Greek Ministry of Education. Informed consent for research involving human participants Parents completed informed consent forms for their participation in the study.
