# Supplementary material for: Network analysis investigating the differentiation of achievement goal orientations in junior high school
Source: F1000Res. 2026 Oct 22;14:1237. Originally published 2025 Nov 10. [Version 2] doi: 10.12688/f1000research.167284.2 (PMC13125999; doi:10.12688/f1000research.167284.2)
Supplement: Peer review history

# Reviewer report: Reviewer response for version 1

**DOI:** 10.5256/f1000research.184380.r436273

**Published:** 2026-04-28

**Authors:** Sudarto M Abukasim

**Affiliations:** 1 Muhammadiyah University of North Maluku, Ternate, Indonesia

This article is considered to make an important contribution to the study of academic motivation and the application of dynamic systems approaches to understanding achievement goals. Overall, the article demonstrates solid scientific quality and has strong potential to enrich the existing literature. Therefore, the manuscript can be accepted, but only pending substantial revisions that need to be addressed before final indexing.

 Points for revision that the authors must address:

  

1. The methodology is too brief to allow replication.
  The Methods section does not provide sufficient detail for other researchers to replicate the study. The authors should elaborate on the ecological momentary assessment procedure, including the frequency of measurements, duration of data collection, and the specific steps taken during data processing.
2. Operationalization and instruments are insufficiently described.
  The measurement of key variables needs to be clarified. The authors should provide more information about the scales used, the number of items, example items, the source of each instrument, and evidence of reliability and validity.
3. Justification for the network model selection is weak.
  The rationale behind the chosen network analysis model, the algorithms applied, and the parameters used must be explained more transparently. The authors should also include information about model stability checks, such as bootstrapping or case-drop analysis, where appropriate.

Is the work clearly and accurately presented and does it cite the current literature?

Yes

If applicable, is the statistical analysis and its interpretation appropriate?

Yes

Are all the source data underlying the results available to ensure full reproducibility?

Yes

Is the study design appropriate and is the work technically sound?

Yes

Are the conclusions drawn adequately supported by the results?

Yes

Are sufficient details of methods and analysis provided to allow replication by others?

Yes

Reviewer Expertise:

Education

I confirm that I have read this submission and believe that I have an appropriate level of expertise to confirm that it is of an acceptable scientific standard.

# Reviewer report: Reviewer response for version 1

**DOI:** 10.5256/f1000research.184380.r436269

**Published:** 2026-01-02

**Authors:** Starr Clyde Lumanta Sebial

**Affiliations:** 1 J.H. Cerilles State College, Pagadian City, Philippines

- The methodology presents **three distinct sets of network analyses**: (a) item-level networks including indicators of six achievement- and teacher-goal constructs; (b) item-level networks with the addition of **self-efficacy** as a seventh construct; and (c) construct-level networks based on **averaged indicator scores** representing the seven variables.
- While each network representation is methodologically sound, the manuscript does **not explicitly explain the analytic or theoretical rationale** for estimating these networks in a sequential manner.
- In particular, it remains unclear:
  - why the initial networks excluded self-efficacy and what baseline structure this was intended to establish;
  - what specific analytic purpose was served by introducing self-efficacy in a separate network stage; and
  - how the construct-level (averaged) networks are intended to complement, validate, or abstract from the item-level networks.
- Given that **node definition and level of aggregation are central ontological decisions in network psychometrics**, clarifying these choices is essential for proper interpretation of the results.
- The authors are encouraged to briefly articulate the **intent of each network set** and to explicitly link each representation to the study’s research questions and theoretical framework, which would substantially enhance methodological transparency and coherence.

Is the work clearly and accurately presented and does it cite the current literature?

Yes

If applicable, is the statistical analysis and its interpretation appropriate?

Yes

Are all the source data underlying the results available to ensure full reproducibility?

Yes

Is the study design appropriate and is the work technically sound?

Yes

Are the conclusions drawn adequately supported by the results?

Yes

Are sufficient details of methods and analysis provided to allow replication by others?

Partly

Reviewer Expertise:

advanced educational statistics, multivariate, structural equation modeling, network analysis...

I confirm that I have read this submission and believe that I have an appropriate level of expertise to confirm that it is of an acceptable scientific standard.
